# Physical Activity and Cognition in Older Adults

**DOI:** 10.1093/geroni/igaf122.266

**Published:** 2025-12-31

**Authors:** Yurun Cai

**Affiliations:** University of Pittsburgh, Pittsburgh, Pennsylvania, United States

## Abstract

Physical activity and exercise in older adults are associated with cognition in older adults. The results of these studies provide insight into this relationship.

